# Procaine–The Controversial Geroprotector Candidate: New Insights Regarding Its Molecular and Cellular Effects

**DOI:** 10.1155/2021/3617042

**Published:** 2021-07-31

**Authors:** Daniela Gradinaru, Anca Ungurianu, Denisa Margina, Maria Moreno-Villanueva, Alexander Bürkle

**Affiliations:** ^1^Department of Biochemistry, Faculty of Pharmacy, Carol Davila University of Medicine and Pharmacy, RO-020956 Bucharest, Romania; ^2^Department of Sport Science, Human Performance Research Centre, University of Konstanz, D-78457 Konstanz, Germany; ^3^Department of Biology, Molecular Toxicology Group, University of Konstanz, D-78457 Konstanz, Germany

## Abstract

Since its discovery in 1905 and its employment in everyday medical practice as a local anesthetic, to its highly controversial endorsement as an “anti-aging” molecule in the sixties and seventies, procaine is part of the history of medicine and gerontoprophylaxis. Procaine can be considered a “veteran” drug due to its long-time use in clinical practice, but is also a molecule which continues to incite interest, revealing new biological and pharmacological effects within novel experimental approaches. Therefore, this review is aimed at exploring and systematizing recent data on the biochemical, cellular, and molecular mechanisms involved in the antioxidant and potential geroprotective effects of procaine, focusing on the following aspects: (1) the research state-of-the-art, through an objective examination of scientific literature within the last 30 years, describing the positive, as well as the negative reports; (2) the experimental data supporting the beneficial effects of procaine in preventing or alleviating age-related pathology; and (3) the multifactorial pathways procaine impacts oxidative stress, inflammation, atherogenesis, cerebral age-related pathology, DNA damage, and methylation. According to reviewed data, procaine displayed antioxidant and cytoprotective actions in experimental models of myocardial ischemia/reperfusion injury, lipoprotein oxidation, endothelial-dependent vasorelaxation, inflammation, sepsis, intoxication, ionizing irradiation, cancer, and neurodegeneration. This analysis painted a complex pharmacological profile of procaine: a molecule that has not yet fully expressed its therapeutic potential in the treatment and prevention of aging-associated diseases. The numerous recent reports found demonstrate the rising interest in researching the multiple actions of procaine regulating key processes involved in cellular senescence. Its beneficial effects on cell/tissue functions and metabolism could designate procaine as a valuable candidate for the well-established Geroprotectors database.

## 1. Introduction

Procaine was synthesized by Alfred Einhorn in 1905 and introduced in clinical practice as Novocain, soon becoming a local anesthetic prototype. Around the 1950s, a large number of accumulated data emphasized the surprising diversity of nonanesthetic effects exerted by procaine, which came to the attention of various medical research schools in Eastern and Western Europe, many doctors exploring, regardless of borders, the beneficial properties of procaine: Vishnevsky and Speransky (Russia); Huneke and Lüth (Germany); Leriche, Dos Ghali, and Hazard (France); Danielopolu and Parhon (Romania) [1–9]. Between 1946 and 1956, Ana Aslan described a significant number of procaine beneficial actions exerted on cellular functions and metabolism, following long-term treatment in low doses, highlighting its “rejuvenating” effects, and developed Gerovital H3 (GH3)—an original procaine-based pharmaceutical formulation [10–12]. Due to these findings, procaine which was known only for its anesthetic properties became one of the most disputed medical developments of the sixties and seventies in the field of “anti-aging” therapy [13–15].

While aging *per se* seems to be the predominant risk factor for most diseases that limit healthspan, the human lifespan can be viewed as a series of gene-environment interactions that inevitably lead to an earlier or later onset of aging-related conditions such as type 2 diabetes, atherosclerosis and cardiovascular diseases, depression, neurodegeneration and cognitive decline, cancer, sarcopenia, osteoarthritis, and osteoporosis [16, 17].

Recent progresses in the field of aging research led to the development of a new class of drugs—geroprotectors, with the ability to target fundamental mechanisms of aging common to multiple age-related diseases, such as response to oxidative damage, inflammation, hypermethylation, cellular senescence, and autophagy [18]. Moskalev et al. (2015) established the first public database of geroprotectors (http://geroprotectors.org) that indexes the most relevant experiments involving over 200 well-established geroprotectors or possible candidates that could extend the healthy lifespan and repair or reduce aging-related damage in model organisms [19]. As primary selection criteria for the potential geroprotectors, the following characteristics were recognized: (1) the ability to increase lifespan; (2) the capacity to ameliorate molecular, cellular, and physiological biomarkers to a younger state or slow the progression of age-related change of these markers; (3) a therapeutic lifespan-extending dose of geroprotector, which should be several orders of magnitude less than the toxic dose; and (4) the capacity to improve the health-related quality of life of the patient, from a physical, mental, emotional, and social viewpoint [20]. The compliance of procaine with most of these criteria would allow it to be a potential “geroprotector” candidate.

Although GH3 was internationally launched in 1956, simultaneously with the development of the Free Radical Theory of Aging by Denham Harman [21], the study of the antioxidant action of procaine and GH3 was documented only after 1980, in various experimental designs, which proved its capacity of limiting the generation of reactive oxygen species (ROS) and lipid peroxidation [22–29]. Recently, the radioprotective effects of procaine and GH3 were reported *in vitro* in human lymphocytes isolated from young and aged individuals [29]. Besides its antioxidant, cytoprotective, anti-inflammatory, and antiatherogenic effects, at cellular and molecular levels, procaine has multiple targets, supporting a large number of potential “geroprotective” effects [30, 31]. Older and more recent data revealed that procaine and its metabolites modulate several biochemical and cellular processes like mitochondrial structure and function [32–34], cholesterol biosynthesis [35], monoamine oxidase (MAO) activity [36, 37], and DNA methylation [38–41].

Procaine is part of the history of medicine and gerontoprophylaxis, an old-timer of clinical practice, but still a molecule with great potential which continues to reveal new biological and pharmacological effects within novel experimental approaches. Therefore, the aim of this review is to explore and systematize data on biochemical, cellular, and molecular mechanisms involved in the antioxidant and alleged geroprotective actions of procaine and GH3, focusing on the following aspects: (1) the research state-of-the-art, through an objective examination of scientific literature for the last 30 years—describing both positive and negative research outcomes; (2) the experimental data supporting the beneficial effects of procaine in preventing the age-related pathology; and (3) the multitude of ways procaine impacts oxidative stress, atherogenesis, cerebral age-related pathology, and DNA methylation.

## 2. Procaine and Gerovital H3—From Anesthetic to “Anti-Aging”

As early as 1892, the German chemist Alfred Einhorn began to model on the structural formula of cocaine, an alkaloid extracted from the leaves of *Erythroxylum coca* and the first known local anesthetic, in order to obtain less addictive molecules, but with similar or enhanced anesthetic qualities. Thus, he synthesized procaine—the first injectable anesthetic, introduced in medical practice under the trade name of *Novocaine*, which means “new cocaine,” from the Latin *nov*- “new” and -*caine*, a common ending for alkaloids used as anesthetics [42].

Therapeutic effects pointed out after systemic administration and anesthetic properties, illustrate different aspects of procaine pharmacodynamics, being determined by dosage and administration routes. It was known by then that the systemic administration of procaine at high concentrations leads to a local anesthetic effect. Of all the local anesthetics, procaine was the least toxic [43–45]. In 1949, the physiologists Danielopolu and Simionescu highlighted procaine's multiplicity of actions, stating that it “exerts uniform action in the organism, restores and enhances active vital processes and local resistance” [8]. Among the researchers who took a close look at procaine, Ana Aslan noticed another quality of procaine: its geroprotective properties. In 1951, she began treating a group of selected patients with 2% procaine, reporting several “rejuvenating” effects in elderly patients: memory enhancement, alleviation of depression, hair repigmentation, better skin tone, and an overall improvement of their condition [10, 11]; all these observations resulted, at that stage, in regarding procaine as “a useful prophylactic and therapeutic substance in the fight against old age” [9, 12]. At the same time, due to its short-term anesthetic effect and need for repeated administration to achieve longer anesthesia, observations regarding the interesting changes occurring following prolonged use started to emerge in the medical community [46]. Aslan sought to alter procaine pharmacokinetics in order to increase its stability in the body, prolong its action and explore its full therapeutic potential. In this purpose, in 1956, a pharmaceutical formulation containing 2% procaine hydrochloride, 0.12% benzoic acid, 0.10% potassium metabisulphite, and 0.01% disodium phosphate (as excipients and stabilizers), with a pH of 3.3, was designed and marketed as GH3 [12, 30]. Pharmacokinetic studies revealed that serum procaine levels are higher after the administration of GH3 than following the one of a procaine solution of similar concentration [47, 48]. Although the studies regarding the effects of GH3 were developed within a large prophylaxis campaign and there were clinical trials involving thousands of elderly subjects, some in the medical world of the 1960s contradicted the so-claimed beneficial effects of the treatment developed by Aslan [13]. The negative outlook and backlash were caused because, at that time, behind Gerovital was more marketing for a “miraculous anti-aging product” than indisputable scientific evidence. In 1982, following the study commissioned by the National Institute on Aging, the U.S. Food and Drug Administration (FDA) banned GH3 for “anti-aging and associated claims” [13–15].

## 3. Procaine Pharmacokinetics and Metabolism

**Figure 1:**
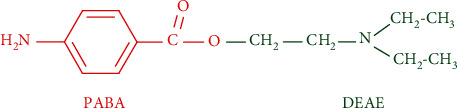
Chemical structure of procaine, ester of *para*-aminobenzoic acid (PABA) with diethyl-amino-ethanol (DEAE).

Procaine is a drug with limited distribution and tissue uptake and a short duration of action: during a continuous intravenous infusion of 2% procaine, the steady-state plasma level is achieved within 20 to 30 minutes. Following the termination of the administration, drug concentration decreases rapidly, with a distribution half-life (t1/2 alpha) of 2.49 ± 0.36 minutes and an elimination half-life (t1/2 beta) of 7.69 ± 0.99 minutes [49]. At systemic level, procaine is hydrolyzed to diethyl-amino-ethanol (DEAE) and *para*-aminobenzoic acid (PABA), as primary metabolites, by the enzyme pseudocholinesterase [50] (Figure 1). In different organs, procaine is hydrolyzed under the microsomal carboxylesterases [51, 52]. DEAE displays local anesthetic activity [53].

Procaine hydrolysis represents an important feature that could support some of its effects on cellular functions and metabolism, as the primary and secondary metabolites could have additional pharmacologic actions or participate as precursors in the synthesis of essential biomolecules. Kietzmann and Kaemmerer (1989) tested the influence of orally-administrated procaine hydrochloride on intermediary metabolism in rats and pointed out the fact that the ratio of acetyl coenzyme A to coenzyme A clearly was enhanced in the liver and, to a minor extent, in the cerebellum. Also, procaine hydrochloride, GH3, as well as DEAE increased, in a dose- and time-dependent mode, the hepatic incorporation rate of amino acids in protein, while PABA yielded no effect [54].

A very attractive hypothesis, which needs more studies and scientific evidence, is the second stage of procaine metabolism (remarkable from the pharmacodynamics viewpoint): the possible generation of ethanolamine from DEAE. Ethanolamine is a possible precursor in the biosynthesis of membrane phospholipids (phosphatidylethanolamine and phosphatidylcholine), which can be converted into the neurotransmitter acetylcholine (Ach) [55–57].

## 4. Aging, Age-Related Diseases, and Antioxidant Action of Procaine and GH3

The “Free Radical Hypothesis of Aging” was put forward 65 years ago, being later revised to the theory known as the “Oxidative Stress Hypothesis” [21, 58, 59]. According to these theories, oxidative stress is caused by the imbalance between the reactive oxygen species (ROS) production and the biological system's ability to counteract, with an appropriate antioxidant defense, resulting in the oxidative damage of cell membranes and other structures such as lipids, lipoproteins, proteins, and DNA [60]. Multiple endogenous sources such as xanthine oxidase, NADPH oxidase, and the mitochondrial respiratory chain can be involved in ROS generation. A variety of environmental stimuli, such as radiation, pathogen infections, and exposure to xenobiotics, can also enhance *in vivo* ROS production [59].

Since many age-related diseases/geriatric syndromes are associated with oxidative stress, and the consequent cellular damage, limiting its intensity became a major area of interest and a common therapeutic target of aging-related pharmaceutical research [61]. Among the strategies of disease prevention and geroprotective therapies, antioxidants are currently still of cutting-edge interest. New experimental approaches, along sensitive and specific methods, are employed in testing and evaluating the actions of natural antioxidant compounds or drugs on alleviating oxidative stress under biological conditions similar to those existing *in vivo* [62, 63].

The antioxidant action of procaine and GH3 has been supported by *in vitro* and *in vivo* studies, in different research models demonstrating the inhibition of ROS generation and lipid peroxidation, associated with a modulating effect on antioxidant enzymes and nonenzymatic antioxidants.

### 4.1. ROS Generation and Lipid Peroxidation—*In Vitro* Studies

In 1989, Rusu and Lupeanu demonstrated for the first time the antioxidant action of procaine, but also of the other GH3 ingredients, namely, potassium metabisulphite and benzoic acid. The inhibitory effect of these products on ROS generation was evaluated *in vitro* using Nishikimi's electron carrier system generating the superoxide radical (O_2_^.–^), comprising reduced nicotinamide adenine dinucleotide (NADH), phenazine methosulfate (PMS), and nitroblue tetrazolium (NBT). GH3 and procaine inhibited the generation of O_2_^.–^ in the presence of increasing concentrations of procaine hydrochloride equivalents (0.2, 0.4, 0.6, 0.8, 1.0, and 2.0 mM), compared to Cu/Zn-superoxide dismutase (SOD)—the antioxidant enzyme that detoxifies superoxide physiologically. The strongest antioxidant effect (68% inhibition of NBT reduction) was exerted at 2.0 mM GH3 [22].

It was proposed that toxic O_2_ metabolites generated by xanthine oxidase (XO) contribute *in vivo* to the development of ischemia-reperfusion injury in a variety of tissues [59]. Gradinaru et al. (2009) examined the antioxidant effects of procaine and GH3 with regard to O_2_^·–^ generation in a physiologic enzymatic system: xanthine – XO – 2-(4-iodophenyl)-3-(4-nitrophenol)-5-phenyltetrazolium chloride (INT). GH3 inhibited INT reduction in a dose-dependent manner, at degrees comparable to SOD. At 10 mM, the maximum inhibition of O_2_^.–^ generation was achieved by GH3 (62%), whereas procaine hydrochloride had a slight (5%) inhibitory effect [25].

Previously, Jinnouchi et al. (2005) studied whether local anesthetics inhibit the priming of neutrophils induced by lipopolysaccharide (LPS). They found that 4.0 mM procaine, 3.0 mM lidocaine, 0.5 mM bupivacaine, or 0.1 mM tetracaine inhibited by 50% the release of O_2_^.–^, in response to triggering by the chemotactic peptide N-formyl-methionyl-leucyl phenylalanine (fMLP) [64]. Librowski and Moniczewski (2010) examined comparatively the antioxidant effect of several local anesthetics. The potency of scavenging radicals, measured as relative scavenging effect (%) of the 2,2′-azino-bis (3-ethylbenzothiazoline-6-sulfonic acid) diammonium salt (ABTS) cation, decreased in the following order: tetracaine > procaine > lignocaine > benzocaine (by 99, 38, 21, and 20%, respectively, at a concentration of 10 mM) [27].

Lee et al. (2010) tested *in vitro* the antioxidant effect of procaine and lidocaine on endothelial-dependent relaxation in the rabbit aorta to examine if their antioxidizing effects could suppress or reduce the ROS-induced endothelial damage. The isolated aortic rings were pretreated with procaine or lidocaine (10^−5^ M to 3 × 10^−3^ M) and subjected to precontraction with phenylephrine (PE). The changes (%) of the aortic tone by acetylcholine (ACh) administration before (control) and after ROS exposure were compared. Their results suggested that procaine and lidocaine dose-dependently preserved endothelium-dependent vasorelaxation against ROS attack, procaine potentially acting via hydrogen peroxide scavenging, as part of its protection mechanism [26]. Takaishi et al. (2013) evaluated the effects of local anesthetics procaine and lidocaine on nitric oxide (NO) production in a bovine aortic endothelial cells culture (BAEC), under proinflammatory conditions. In bradykinin and Ach stimulated cells, 10 mM procaine significantly inhibited NO production by 35%, whereas in cells incubated with interleukin-1 beta (IL-1*β*) and LPS, 10 mM procaine significantly inhibited NO production by 15%. Authors suggested that the inhibitory effects of procaine on NO production are partially due to the suppression of L-arginine uptake [65].

The antioxidant action of GH3 and procaine under proinflammatory conditions was recently tested by Ungurianu et al. (2020) using a human lymphoblastoid cell line as experimental model. Membrane lipid peroxidation in Jurkat cells was induced by cumene hydroperoxide (CuOOH) and assessed with diphenyl-1-pyrenylphosphine (DPPP), a sensitive fluorescent probe. The preincubation of Jurkat cells with 2.5, 5.0, and, respectively, 10 mM procaine or GH3 effectively reduced the generation of cell membrane lipoperoxides, but GH3 was more effective than procaine especially at the lowest concentration (2.5 mM), when GH3 prevented lipid peroxidation by 21%, versus only 5% for procaine. At 5 and 10 mM, procaine and GH3 showed similar patterns of antioxidant action [29].

Ionizing radiation contributes to ROS generation and DNA damage which is known to be one of the mechanisms responsible for increasing mutagenesis risk, vascular aging, cancer, and neurodegenerative diseases [66–69]. The radioprotective effects of procaine and GH3 (0.25, 0.5, and 1 mM) on the formation of endogenous and X-ray-induced DNA strand breaks in peripheral blood mononuclear cells (PBMCs) isolated from young and elderly individuals were recently investigated. Interestingly, at low concentrations (0.25, 0.5, and 1 mM), GH3 showed the strongest radioprotective effects in PBMCs from young subjects, while procaine reduced the endogenous amount of DNA strand breaks more pronounced in aged individuals. Concentrations of procaine and GH3 of 3 mM and higher (5 and 10 mM) showed a genotoxic effect as measured by DNA strand breaks formation, using the automated fluorescence-detected alkaline DNA unwinding (FADU) assay [29].

### 4.2. Mitochondria Function

In recent years, it was reported that mitochondria, besides being the main cellular source of ROS, might also be the most relevant target of free radicals, playing a central role in aging [70]. Mitochondria are particularly prone to lipid peroxidation [71, 72], and there is a strong link between mitochondrial metabolism, oxidant species formation, and the biology of aging [73–75]. Mitochondrial ROS also increases with age, and the oxidative stress-dependent decline of cell functions is partially related to the impairment of the mitochondrial respiratory chain [76–78].

As an anesthetic, procaine binds to membrane constituents and modulates a series of ion channels, interacts with membrane phospholipids, and induces concentration-dependent changes in membrane fluidity [42, 79–81]. Moreover, mitochondria, which are considered the powerhouses of the cell, are a potential target for general and local anesthetics [34]. At the cellular and molecular levels, procaine and its metabolites affect several biochemical and cellular processes like membrane conductance [81], oxidative phosphorylation [32], mitochondrial function and structure [33], or fatty acids oxidation [82].

Following an *in vivo* treatment (1 mg procaine/100 g body weight, for 3 days), procaine facilitated oxygen transport towards the mitochondrial matrix by modifying the membrane structure of brain mitochondria in both old and young rats [32]. The mitochondrial ATP-sensitive potassium channel (mitoKATP) is an important component of the mitochondria, whose opening is caused by a calcium signal or by brief episodes of ischemia-reperfusion [34]. de Klaver et al. (2006) demonstrated in human microvascular endothelial cells that tetracaine and procaine had no protective effect against the cell injury induced by inflammation (LPS) and inhibited the activation of mitoKATP channels [83].

Tarba and Crăcium (1990) pointed out, in isolated rat liver mitochondria, a stimulation of the basal state (respiration before ADP addition) in presence of low concentration (1 mM) of procaine. Moreover, procaine had a biphasic effect, exerting a slight stimulation of state-3 respiration (ADP present) at low and moderate concentrations (≤1 mM) and an inhibition at higher concentrations (>1 mM). Besides, electron microscopy confirmed this inhibitory effect, showing an abundance of either swollen or supercondensed mitochondria, with many membrane ruptures. At very low procaine concentrations (0.01–0.1 mM), the stimulation of the two respiration states is approximately equal and thus the uncoupling effect is absent or negligible [33]. High concentrations (>10 mM) of procaine and GH3 inhibited the uncoupling effect of 2,4-dinitrophenol (2-DNP) on oxidative phosphorylation and stimulated the respiratory activity and induced membrane rigidity thus allowing preferential oxygen diffusion and acceleration of free radical reactions, whereas low concentrations facilitated diffusion of sulfhydryl (SH) containing-compounds, exerting protective effects against lipid peroxidation [84]. Since mitochondrial injury is considered a central event in the early stages of the nephrotoxic effect of the antineoplastic drug cisplatin, Zicca et al. (2002) demonstrated that procaine hydrochloride was able to protect mice and rats through its accumulation in kidneys, followed by coordination with cisplatin (or its hydrolysis metabolites) and formation of a less toxic platinum compound [85]. Previously, Zhang and Lindup (1994) pointed out that procaine (2 mM), DEAE, and PABA protected against the rat kidney cellular damage caused by cisplatin and inhibited by 24, 30, and 22%, respectively, the cisplatin-induced mitochondrial lipid peroxidation, without any changes regarding the mitochondrial protein sulfhydryl groups (protein-SH) [86].

Studies conducted by Onizuka et al. (2010) in rat dorsal root ganglion neurons demonstrated the depolarizing effect of procaine on the mitochondrial membrane by increasing the mitochondrial and intracellular pH, in a dose-dependent manner [87]. Yu et al. (2017) proved that procaine significantly increased neurotoxicity at high concentrations (12, 15, and 20 mM), inducing mitochondrial dysfunction, overproduction of ROS, lipid peroxidation, DNA damage, and apoptosis, in human neuroblastoma cell line SH-SY5Y cells [88].

Recently, using a fluorescent assay with N-acetyl-3,7-dihydroxyphenoxazine (Amplex Red), Ungurianu et al. (2020) showed the inhibitory effect of procaine and GH3 on rat liver mitochondria lipid peroxidation providing additional experimental data concerning their antioxidant action in these biological structures. At any of the employed concentrations (0.5, 1.0, 2.0, 5.0, and 10 mM), procaine tended to inhibit lipid peroxidation at higher levels compared to GH3. The inhibitory effect was substantial at the lowest concentration (0.5 mM), 32% for GH3 and 42% for procaine, and increased in a dose-dependent manner. Both procaine and GH3 inhibited at 10 mM more than 80% of the reactive peroxides production in isolated mitochondria fraction [29].

### 4.3. Cellular Antioxidant Systems—*In Vivo* Studies

A significant number of studies reported the beneficial effects of *in vivo* treatments with procaine and GH3 in animal models, by modulating the expression of antioxidant enzymes. Thus, chronic treatment with GH3 (20 mg/procaine/kg body weight, three times a week, for nine weeks) modulated lipid peroxidation in homogenates from rat brain tissue as well as O_2_^.–^ generation, in correlation with an increase in SOD enzymatic activity [23]. Additionally, procaine and GH3 also induce a significant increase of catalase (CAT) activity in liver and kidney of young, adult, and old rats, and a decrease in the heart of old and adult female rats [89]. Previously, studies using fluorescence and electronic microscopy showed that GH3 treatment decreased lipofuscin **(**the aging pigment**)** accumulation in rat brain, testicles, liver, and heart [90, 91].

Procaine as well as procainamide is usually used for the therapy of cardiac arrhythmias. Recently, Qiang et al. (2019) investigated, *in vitro* and *in vivo,* the protective effect of novel 1,3,5-triazine-procaine derivatives against myocardial ischemia-reperfusion injury on the basis of various parameters, such as hemodynamic indices, myocardial enzymes, oxidative stress biomarkers: antioxidant enzymes (SOD, CAT, and glutathione peroxidase [GPx]), glutathione (GSH), hydroxyl radical and superoxide anion scavenging assays, as well as cardiac histopathological examination. Results showed an efficient reduction of ROS, as well as restored to normal levels of GSH and SOD, CAT, and GPx enzymatic activities in the triazine-procaine derivatives-treated group, as compared to myocardial ischemia-reperfusion group. Within this comprehensive experimental model, procaine-1,3,5-triazine derivatives showed significant cardioprotective action via inhibition of nuclear factor-kappa light chain enhancer of activated B cells (NF-*κ*B) [28].

### 4.4. Lipoprotein Oxidation and Metabolism

Plasma lipoproteins are perfect biological “sensors” of oxidative stress in the arterial wall due to their close interactions with vascular endothelial cells and the high susceptibility of lipids to oxidative alterations [92]. Of particular interest is the impact of oxidative stress on plasma low-density lipoproteins (LDL), as oxidized LDL (oxLDL) are recognized to play a crucial role in promoting atherogenesis by several mechanisms involving their cytotoxicity on monocyte-derived macrophage cells, through reactive species generation and antioxidant failure [93, 94]. Therefore, LDL are the most targeted by oxidative stress associated with metabolic imbalances such as hyperlipidemia, hyperglycemia, or insulin resistance. Increased LDL oxidation and development of a low-grade proinflammatory environment have been proposed to contribute to age-dependent endothelial dysfunction [94].

Gradinaru et al. (2009) studied *in vitro* the effect of procaine and GH3 on LDL oxidation, by incubating native LDL isolated from human plasma with 0.1, 0.5, and 1 mM procaine/GH3. The kinetic analysis of conjugated dienes formation during the Cu^2+^-induced oxidation of LDL revealed that 1 mM procaine significantly inhibited LDL oxidation at 20 and 60 minutes after its inducement, whereas 1 mM GH3 exerted a long-lasting antioxidant effect, with a significant inhibition even after 180 minutes [25].

Ungurianu et al. (2020), using a sensitive-fluorescent assay with Amplex Red, also pointed out, in human serum lipoprotein concentrates, a dose-dependent inhibitory action of procaine and GH3 on lipid peroxidation, significant at all tested concentrations (0.5, 1.0, 2.0, 5.0, and 10 mM). GH3 showed a significantly higher lipid peroxidation inhibition compared to procaine. Additionally, the effect of GH3 and procaine treatment was examined on cell-mediated LDL oxidation induced in human-derived U937 cultured macrophages. Cellular oxidative stress was evaluated using the thiobarbituric acid reactive substances (TBARS) released in the incubation medium, as oxidative stress biomarker for the global measurement of lipid peroxidation end-products such as malondialdehyde (MDA) and 4-hydroxynonenal (4-HNE). At all the tested concentrations (0.5, 1.0, and 2.0 mM), GH3 significantly decreased TBARS (%), whereas the effect of procaine was lower, reaching only half of GH3 effect at 2 mM [29].

Overall, procaine's significant antioxidant action demonstrated by *in vitro* and *in vivo* studies may contribute to its geroprotective effect, but the question is which are the molecular mechanisms for procaine's antioxidant effect? It could act as an inhibitor of the chain reactions generating the lipid peroxides, or/and as “scavenger” of the ROS. While procaine was more effective in protecting the cellular and mitochondrial membranes, GH3 was more efficient against serum lipoperoxidation. These outcomes could be explained through the different lipid or lipoprotein microenvironments present in these biological systems and/or through the different intrinsic antioxidant capacities or ROS scavenging actions of procaine and GH3, in counteracting or preventing lipid peroxidation [29].

The main limitation of these studies is that they present overall effects and did not investigate the specific molecular and cellular mechanisms involved, such as the modulation of signaling pathways which can result in enhancing cell antioxidant response. Therefore, further investigations are needed to establish the exact mechanisms of action of procaine and GH3.

Several experimental studies have highlighted the role of procaine as a modulator of lipid metabolism [95, 96]. The lipid-lowering action of procaine was explained via a “statin-like” action exerted through the regulation of 3-methylglutaryl-coenzyme A (HMG-CoA) reductase, the key enzyme in cholesterol biosynthesis [35]. The effect of procaine on steroidogenesis was reported in human H295R adrenal cells and in procaine-treated rats. This inhibitory activity was not observed in Leydig cells, suggesting that the effect could be specific to adrenocortical cells. Procaine did not affect either cyclic AMP- (cAMP-) dependent protein kinase activity, key proteins involved in mitochondrial cholesterol transport, side-chain cleavage enzymes, or enzymatic activities associated with the final stage of cholesterol biosynthesis. However, procaine reduced HMG-CoA reductase activity and specific mRNA expression dose-dependently. The modulatory effect of procaine on HMG-CoA reductase mRNA was also observed in the Hepa 1-6 mouse hepatoma cells stimulated by dibutyryl-cyclicAMP (dbcAMP) [35].

Procaine was also tested *in vitro* for its action as an inhibitor of the two enzymes involved in cholesterol esterification: acyl-CoA cholesterol acyltransferase (ACAT) and lecithin-cholesterol acyltransferase (LCAT) [97, 98]. Bell and Hubert (1981) used a microsomal fraction isolated from rabbit aorta, in which they monitored the incorporation of [^14^C]-oleyl-SCoA in the form of [^14^C]-cholesterol esters. The ACAT activity was inhibited depending on the concentration of anesthetic (0.25-0.50 mM procaine) in the reaction medium [97]. Another experiment was performed *in vitro* in human, rat, and dog plasma samples incubated with 1-5 mM procaine, which significantly inhibited LCAT activity [98]. Some longitudinal and clinical studies carried out in elderly subjects with systemic atherosclerosis evidenced the lipid-lowering and antioxidant actions of GH3 treatment [24, 30].

These results lead to a complex pharmacological profile of procaine, which includes the modulation of cholesterol metabolism at all levels, from genetic control of sterol biosynthesis to its esterification in plasma and tissues, with potential clinical applications in the treatment of hypercholesterolemia. Moreover, elevated glucocorticoid levels are associated with many pathologies, including age-related depression, hypertension, Alzheimer's disease (AD), or acquired immunodeficiency syndrome, cortisol biosynthesis reducing agents being a possible useful complementary therapy for all these conditions.

All these data may suggest that a combination of GH3 with lipid-lowering drugs could diminish the doses and the adverse effects of the classical treatment of hyperlipidemia.

## 5. Cerebral Age-Related Pathology

Similar to the structurally related cocaine, the effects of procaine go beyond its anesthetic actions, with some concerning the central nervous system (CNS). Several beneficial effects of procaine and GH3 were reported, such as ameliorating depression and cognitive abilities (conditioned behavior, memory) and increasing cerebral resistance to different aggressive actions (acute intoxication, hypoxia, and electric shock) [36, 37, 99–105].

Monoamine oxidases (MAOs), a class of enzymes involved in the metabolism of catecholamines and other biogenic amines, are increasingly recognized as major contributors to the generation of mitochondrial ROS. The best-known and characterized MAOs are the endothelial and neuronal ones. However, the inducible isoforms which can be expressed in various tissues and organs have lately gained notoriety and stimulated interest in the extracerebral roles of these enzymes. For an overview of the complex roles of MAOs in age-associated diseases, the reader is referred to a recent review by Santin et al. (2021) [106]. It is well known that the expression of human MAOs and their abilities to produce ROS increase with age (4-fold MAO-B in neuronal tissue and 6-fold MAO-A in the heart) and are involved in the etiology of age-associated chronic pathologies: depressive disorders, Parkinsonism, cardiac diseases, and diabetes [107, 108].

In 1940, Philpot evidenced for the first time, using rat liver homogenate, procaine's inhibitory effect on the tyramine and adrenaline oxidation, at high concentrations (33 mM procaine) in the reaction mixture [109]. Further studies pointed out MAO inhibitory actions for both procaine and GH3 in brain, liver, and heart tissues from mice and rats [110, 111]. The MAO B inhibitory action of procaine was reported following pharmacodynamics studies, and GH3 was included in the category of reversible and competitive inhibitors [112, 113]. The inhibition of MAO activity by local anesthetics depends on both electrostatic and hydrophobic interactions between these drugs and enzyme-associated phospholipids or the hydrophobic regions of proteins [114]. MAO inhibitory effect was associated with the inhibition of lipid peroxidation in rat brain homogenates and mitochondrial fraction, pointing out that MAO activity could be inhibited through a limitation of free radical reactions [115].

Using rat pheochromocytoma PC12 cells, Lecanu et al. (2005) observed that procaine is a ligand of the sigma 1 receptor, a protein whose ligands have been shown to protect mitochondrial function and to exert antidepressant properties. Procaine also displayed strong neuroprotective properties against the amyloid peptide A*β*_1-42_ and preserved A*β*_1-42_-induced ATP depletion. Procaine inhibited the neurotoxic effect of glutamate on PC12 cells, suggesting that the reduction of glutamate-induced neurotoxicity may be the mechanism by which procaine exert its “anti-amyloid” effect [116]. Li et al. (2016) studied the effect of procaine treatment in a rat model of neuropathic pain. Procaine inhibited Janus kinase 2 (JAK2) and signal transducer and activator of transcription 3 (STAT3) expression, both mRNA and protein levels, indicating its cellular mechanism in attenuating neuropathic pain [117]. Recently, Wu et al. (2020) synthesized a series of procaine-imidazole derivatives with potent and selective MAO-B inhibitory activity, as well as *in vivo* anti-Parkinson effects using a 1-methyl-4-phenyl-1,2,3,6-tetrahydropyridine (MPTP) model of Parkinson's disease. These procaine-based compounds determined a significant improvement of motor function in mice as revealed by motor behavioral assessment using the footprint and horizontal wire test and also improved the level of antioxidant enzymes in the striatum of animal brains [37].

Older studies regarding the effects of GH3 treatment on the function of CNS reported an improvement of cognitive function, better work capacity, increased resistance to stress and effort, decrease of depression symptoms, and an overall higher capacity of adapting to external stimuli. These data underlined that geriatric procaine-based products have minimum/short-lived side effects compared to classic CNS-targeted medication, tolerated with difficulty by elderly with polypathology [99, 100, 110, 118, 119]. For decades, procaine infusions have been applied in patients with psycho-vegetative disturbances, mostly during neural therapy—a common complementary treatment approach using injections with short-acting local anesthetics to treat pain and chronic diseases. However, little is known about the underlying mechanisms and the domains of treatment response [102, 103].

Hahn-Godeffroy et al. (2019) studied, in 56 case-control patients, the effect of intravenous procaine (1-3 ampoules of 5 ml of procaine 2% in 250 ml sodium chloride per treatment setting) on the somatic and psycho-vegetative state of health. After 4 or 6 months, 75% of patients showed an improvement in the 9 positive items, e.g., “hedonia,” “joyness,” or “improved sleep.” 62.5% of patients reported a substantial attenuation in the 12 negative items, e.g., “stress reactions,” “loss of energy,” or “anxiety.” All these changes were significant after 2, 4, and 6 months compared to the values at baseline, suggesting a long-lasting improvement of somatic and psycho-vegetative symptoms under the infusion of procaine alone, which modulates the activities of specific brain areas such as the limbic system [102].

Haller et al. (2018) performed, in 22 patients with multiple diagnoses, a qualitative analysis of self-reported outcomes following neural therapy injections with procaine. Patients experienced an emotional release and physical symptoms relief, consisting of improved mood, increased pain acceptance, and empowerment. Adverse events of neural therapy included pain at the injection site, vegetative complaints, and emotional turmoil that lasted for minutes or hours, with a maximum of two days [103]. Oettmeier et al. (2019) reported the clinical use of a highly-dosed infusion of procaine hydrochloride with sodium bicarbonate as an additive, for the treatment of different acute diseases, chronic pain, and inflammations [31].

Xu et al. (2016) explored the effects of daily use of GH3 tablets, for three months, on relieving mental symptoms and improving health-related quality of life among Chinese older adults. The randomized, placebo-controlled, double-blinded study comprised 100 eligible participants, men and women between 50 and 89 years of age. GH3 treatment showed positive outcomes in supporting mental health and improving general health and well-being, while promoting the recovery of cognitive function among older adults. Average levels of low mood and anxiety concerns (evaluated with Self-Rating Depression and Anxiety Scales) were both reduced, and the prevalence rate of clinical anxiety was decreased [104].

## 6. Negative Results Reported in Experimental and Clinical Studies

The use of procaine for nonlocal-anesthetic purposes is highly controversial, especially when employed for its alleged “anti-aging effects” [15]. Different pharmaceutical preparations, including GH3, were widely promoted and commercially available “over the counter” in any country, especially via online marketing. Also, there are reports of studies which comparatively examined the effects of procaine (and GH3) and other anesthetics in different age-related maladies reported a lack of efficacy, as well the presence of toxicity or even severe side-effects [13, 14, 120–123].

Several experimental and clinical studies described significant neurologic alteration following procaine and GH3 treatment. In a review commissioned by the National Institute on Aging of the National Institutes of Health (USA), Ostfeld et al. (1977) evaluated scientific literature on the systemic use of procaine in the treatment of the aging process and the common chronic diseases, including data from 285 articles and books, describing the treatment in more than 100,000 patients. Except for a possible antidepressant effect, they found no convincing evidence that procaine or GH3 has any value in the treatment of aging-associated diseases in older patients [13] In a systematic review, Szatmari and Bereczki (2008) assessed independently the efficacy and adverse effects of procaine and GH3 treatment regarding cognitive function improvement in subjects with dementia from randomized, double-blind trials carried out before the 1990s. Pooling data from two studies showed a detrimental effect of procaine in terms of causing side effects. In patients with dementia, a single small study also suggested a negative effect, while two trials including healthy elderly individuals suggested a positive effect of procaine use on cognitive function. Authors concluded that the evidence for detrimental effects of procaine and its preparations is stronger than the data reporting its benefits in preventing and/or treating dementia or cognitive impairment [14].

Zaric and Pace (2009) searched the Cochrane Central Register of Controlled Trials for frequency of transient neurologic symptoms (TNS—painful condition that occurs in the immediate postoperative period) and neurologic complications after spinal anesthesia with lidocaine compared to other local anesthetics, including procaine. The risk of developing TNS after spinal anesthesia with lidocaine was significantly higher than when bupivacaine, prilocaine, or procaine were used [123]. Ghafari et al. (2012) evaluated, in a prospective, randomized, double-blind trial, comprising 110 patients (aged between 20 and 70 years), and the effect of lidocaine versus procaine on cognitive impairment manifested after coronary artery surgery. In the procaine group, the neurocognitive total score decreased significantly compared to the preoperative score and compared to the lidocaine group [122].

Takenami et al. (2012) aimed to compare the neurotoxicity of intrathecal procaine, bupivacaine, levobupivacaine, and ropivacaine in a rat spinal model. Although the four local anesthetics seemed to cause identical neurotoxic lesions commencing in the posterior root and extending to the posterior column by axonal degeneration, bupivacaine appeared to be the most neurotoxic of the four drugs, and the neurotoxicity at higher doses increased by administered volume with procaine > levobupivacaine > ropivacaine [121]. Yilbas et al. (2018) studied the effect of intra-articular procaine injection on knee articular cartilage and the synovium of Sprague-Dawley rats. Results showed no significant differences in inflammation (using a histological evaluation) between procaine and saline (control) groups at any duration of treatment (after 1, 2, 7, 14, and 21 days). No significant difference was detected in the percentage of apoptotic chondrocytes between groups at any of the time intervals [120].

## 7. DNA Methylation and Tumorigenesis

DNA methylation is an epigenetic modification involved in gene expression regulation. Age-associated alterations in DNA methylation are commonly grouped in the phenomenon known as “epigenetic drift,” which is characterized by gradual extensive demethylation of genome and hypermethylation of a number of promoter-associated 5′-cytosine-phosphate- guanine-3′ (CpG) islands. For an overview on the reconfiguration of DNA methylation in aging the reader is referred to a recent review by Zampieri et al. (2015) [124]. Possible consequences are mutations and dysregulation of gene expression, which can either lead to cell death or cellular senescence or to malignant transformation of the cells, ultimately resulting in cancer [125]. DNA methyltransferases (DNMTs) are a family of enzymes that methylate DNA at the C5 position of cytosine followed by a guanine residue (CpG dinucleotide). Reexpression of methylation silenced tumor suppressor genes by inhibiting the DNMTs (DNMT1, DNMT3A, and DNMT3B) has emerged as an effective strategy against cancer [126]. A large number of preclinical studies have shown that local anesthetics have a direct inhibitory effect on tumor activities, including cell survival, proliferation, migration, and invasiveness [41, 127, 128]. Recently, Moreira-Silva et al. (2020) cited procaine among the “repurposed drugs” which have demonstrated promising results as epigenetic inhibitor in *in vitro* tumorigenesis [129].

DNA hypermethylation and the consequent silencing of tumor suppressor genes are considered as a molecular hallmark of many kinds of cancers. Villar-Garea et al. (2003) demonstrated for the first time the role of procaine as a DNA demethylating agent in breast cancer cells, evidencing a 40% reduction in 5-methylcytosine (5mC) DNA content. Procaine had also the capacity to demethylate densely hypermethylated CpG islands, such as those located in the promoter region of the retinoic acid receptor (RAR) beta2 gene, restoring gene expression of epigenetically silenced genes. Finally, procaine also had growth-inhibitory effects in these cancer cells, causing mitotic arrest [38]. Gao et al. (2009) provided the first evidence that procaine is able to reactivate, in lung cancer cells, the Writ inhibitory factor-1 (WIF-1), which was silenced due to promoter hypermethylation [130].

Using hepatoma cells and nude mice bearing xenograft, Tada et al. (2007) revealed that procaine displayed both growth-inhibitory and demethylating effects on human hepatoma cells, both *in vitro* and *in vivo*. All the genes transcriptionally suppressed by DNA hypermethylation were demethylated and reactivated following procaine treatment. Morphological observations showed a significant reduction in tumor volume *in vivo* [131]. Castellano et al. (2008), who synthesized several analogues of procaine and tested their inhibiting activity against DNMT1, discovered a derivative able to induce a recognizable demethylation of chromosomal satellite repeats in HL60 human myeloid leukemia cells [132].

Another mechanism underlining procaine's anticancer activities is through direct interaction with DNA. In a multispectroscopic and molecular modelling study, Ali et al. (2018) used molecular docking on five different B-DNA structures (extracted from the Protein Data Bank) and showed that procaine binds to the adenine-thymine (AT) rich region of all five calf thymus B-DNA structures. Simultaneously, they found that procaine acts as an electron donor to DNA bases when testing the anticancer activity of procaine alone and in combination with doxorubicin in MCF-7 breast cancer cells [133].

Procaine also demonstrated nonepigenetic effects, such as the inhibition of cell proliferation and migration, and also enhancement of apoptosis in gastric cancer cells, osteosarcoma cells, colon cancer cells, mouse models of lung cancer, human leukemia cells, and human bladder cancer cells [134–139]. Borutinskaite et al. (2016) examined the effects of procaine as DNMT inhibitor on growth inhibition, apoptosis, and differentiation of human leukemia cells and showed an increase in the expression of molecules associated with differentiation, such as integrin CD11b, E-cadherin, granulocyte colony-stimulating factor (G-CSF), and apoptosis-peroxisome proliferator-activated receptor (PPAR) gamma. Moreover, procaine enhanced certain gene transcription activation via chromatin remodeling—the changes in histone H3K4 (Me)3 and H3K9Ac/S10P modifications were detected [138]. Sun et al. (2012) proved that procaine might be used as a potential agent for bladder cancer treatment as it inhibited the proliferation of T24 and 5637 human bladder cancer cells by inducing their apoptosis. The mechanism studies reveal that procaine could induce demethylation of apoptotic peptidase activating factor 1 (APAF1) gene in T24 or 5637 cells, subsequently activating caspase-3/9. It was also shown that the serum soluble fas ligand (sFasL) was activated, and the expression of matrix metallopeptidase 9 (MMP-9) was downregulated [139]. The common mechanism by which procaine inhibited cancer cell proliferation and migration was the inactivation of the extracellular signal-regulated kinase (ERK)/mitogen-activated protein kinase (MAPK)/focal nuclear adhesion kinase (FAK) and protein kinase B (AKT)/extracellular signal-regulated kinase (ERK) pathways [134, 135]. In a recent study, Fan et al. (2021) identified a novel mechanism through which procaine can impair the survival and self-renewal of the malignant glioblastoma stem cells, suggesting that local anesthetics may weaken zinc finger Asp-His-His-Cys-type palmitoyltransferase 15 (ZDHHC15) transcripts and decrease glycoprotein 130 (GP130) palmitoylation levels and membrane localization, thus, inhibiting the activation of interleukin-1 (IL-6)/signal transducer and activator of transcription 3 (STAT3) signaling [41].

As to procaine's effects in normal cells, Schumann et al. (2020) characterized the action of procaine and S-adenosyl-L-homocysteine (SAH) as demethylating agents, on the expression of genes related to the epigenetic machinery, including the DNMTs and on DNA methylation levels in bovine skin fibroblasts. Global DNA methylation levels were significantly lower in cells that were cultivated in medium containing both compounds versus control cells, and gene expression of DNMT1, DNMT3A, and DNMT3B decreased significantly in cells cultivated with SAH + procaine (1 mM). Moreover, a significant decrease in DNMT3B transcript levels was found in cells cultivated with procaine. Higher levels of the ten-eleven translocation enzyme-3 (TET3) dioxygenase, involved in the epigenetic machinery, were also found in cells cultivated with procaine and SAH + procaine, compared with the control [40].

Using a mouse behavioral sensitization model in which animals were subjected to an acute treatment with procaine for seven days, Anier et al. (2018) found that procaine caused a decrease on the DNMT3A mRNA levels in peripheral blood cells (PBCs), suggesting that the inhibition of voltage-gated sodium channels may be the mechanism that alters DNMT expression in PBCs [39].

## 8. Procaine Effects on Lifespan

The studies researching procaine's influence on lifespan are scarce. An experimental study conducted by Aslan et al. (1965) on 1840 rats pointed out 18-21% longer lifespan in treated animals than that of controls injected with saline solution [140]. Another investigation on lifespan was conducted on 3680 animals from 5 successive generations. The outcome supported that GH3 administered since early ages induced a lifespan extension both in the treated animals, as well as in the first generation of not-treated offspring [141]. In *Drosophila melanogaster* grown on nutritive medium enriched with GH3 was also found out a 22.7% lifespan extension, compared with controls [142]. Unfortunately, there are no recent studies regarding the effects of procaine and/or GH3 on lifespan.

## 9. Conclusions and Perspectives

The analysis of older and more recent (between 1990 and 2020) literature data reveals the diversity of procaine's effects at cellular and molecular levels, in preclinical studies and clinical settings.

Summarizing its cellular actions, procaine is able to bind to membrane constituents and interact with a series of ion channels exerting its anesthetic action [42, 143] and also has a significant influence on oxidative stress response, on the modulation of critical metabolic pathways, as well as on epigenetic regulation.

The antioxidant action of procaine is supported by *in vitro* and *in vivo* experimental studies regarding the inhibition of ROS generation and lipid peroxidation, in enzymatic [25] and nonenzymatic systems [23, 27], associated with a modulating effect on antioxidant enzymes [23, 28, 89]. Several studies confirm its involvement in mitigating cellular and systemic oxidative stress, acting on the main targets of aging and age-related diseases: cell membranes [22, 24], lipoproteins [25, 29], mitochondria [29, 37, 86], and DNA [29]. Procaine reaffirmed its antioxidant and cytoprotective actions in experimental models of myocardial ischemia/reperfusion injury [28, 83], endothelial-dependent vasorelaxation [26], inflammation [65], sepsis [64], ionizing irradiation [29], and intoxication [85, 86] (Table 1).

Like many pharmacologic active molecules, procaine exhibits a multimodal dose response. In low concentrations (≤1 mM), protective effects were reported regarding mitochondrial function, lipid peroxidation, and DNA damage, whereas high concentrations (>10 mM) induced membrane rigidity, acceleration of free radical reactions, genotoxicity, neurotoxicity, mitochondrial dysfunction, and apoptosis [29, 33, 87, 88] (Table 2).

Procaine might exert its actions regarding the atherogenesis process by modulating lipoprotein metabolism, as an inhibitor of the key enzymes involved in cholesterol biosynthesis and esterification: HMG-CoA reductase, ACAT, and LCAT [35, 97, 98], and via its antioxidant mechanisms, reducing the oxidative stress exerted on the LDL [25, 29] (Table 3).

Numerous beneficial actions were reported for procaine concerning the CNS, beyond its anesthetic effect. Experimental studies highlighted neuroprotective, antidepressant, and “anti-amyloid” actions [116], along inhibition of JAK2 and STAT3 expression in neuropathic pain models [117]. The inhibitory effect on MAO-B of procaine-derivatives, as well as the *in vivo* anti-Parkinson effect, was associated with lower levels of mitochondrial lipid peroxidation and improved levels of antioxidant enzymes in the striatum [37] (Table 4).

Various recent clinical studies pointed out the functional improvement of somatic and psycho-vegetative symptoms during neural therapy with procaine [102, 103]. GH3 treatment shows positive effects in supporting mental health and improving general health and well-being, while promoting the recovery of cognitive function among older adults [104].

Procaine could be considered as a reference substance for DNA-demethylation and tumor-suppressive effects, although these interventions may only be detectable in specific types of cancer due to differential methylation profiles [144]. Recently, procaine was included among the potential “repurposed drugs” with promising results as an epigenetic modulator [129]. An important number of preclinical studies demonstrated the role of procaine as DNA-demethylating agent through the inhibition of DNA methyltransferases in normal [39, 139] and cancer cells [132], or through direct interaction with DNA [133]. In a variety of cancer cells, procaine is able to reactivate tumor suppressor genes, such as WIF-1 [130], and impair the survival and self-renewal of the malignant cells by inhibiting the activation of IL-6/STAT3 signaling [41]. Procaine also inhibits cancer cell proliferation and migration, enhancing apoptosis, through the inactivation of the ERK/MAPK/FAK and AKT/ERK pathways [134–139, 145] (Table 5).

**Figure 2:**
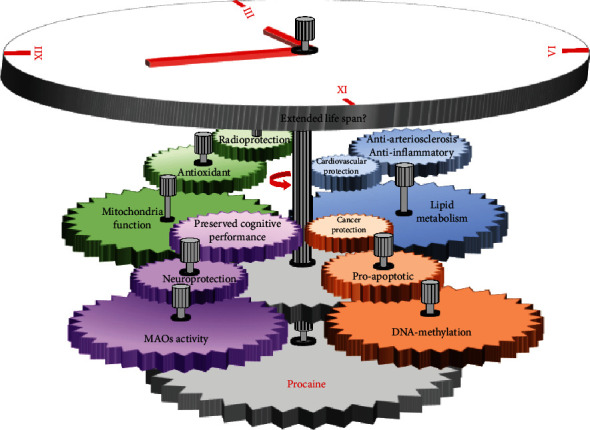
New biological and pharmacological effects of procaine—demonstrated within novel experimental approaches—which could acknowledge its consideration as a potential geroprotector candidate.

In conclusion, beyond its well-known anesthetic action, procaine displays a variety of biological and pharmacological effects, functioning as an antioxidant, anti-inflammatory, cardioprotective, neuroprotective, radioprotective, cytoprotective, and demethylating agent. The beneficial effects on cellular functions and metabolism could designate procaine as a valuable candidate for the Geroprotectors (http://geroprotectors.org) database (Figure 2).

Future research approaches are likely to evaluate procaine's effects in animal and cellular experimental models, focusing on lifespan assessment, autophagy and proteasome regulation, replicative senescence—telomere length and telomerase activity, cell cycle regulation including aging-related pathways such as insulin/insulin-like growth factor 1 (IGF-1)/phosphatidylinositol-3 kinase (PI3K)/AKT (Protein Kinase B) and the Forkhead box O (FOXO) transcription factors (FOXOs), as well as the energy sensing molecular apparatus comprising the mammalian target of rapamycin (mTOR), the adenosine monophosphate-activated protein kinase (AMPK), and the sirtuins. Only after extended investigation with novel experimental approaches, we will be able to fully understand the modulatory action of procaine in the mechanisms of aging and the etiology of chronic degenerative maladies. Following the in-depth comprehension of the fascinating multiples facets of procaine, a judicious use of procaine-based drugs could be employed in the prophylaxis and treatment of different metabolic and degenerative disorders commonly encountered in elderly patients, but which nowadays seem to affect younger and younger individuals.

## Figures and Tables

**Table 1:** Molecular and cellular effects of procaine reported within *in vitro* and *in vivo* studies, which support its antioxidant action.

Preclinic model	Target	Concentrations/doses	Relevant finding	Reference
*In vitro* superoxide (O_2_^.-^) generation	Nonenzymatic system: [NADH-PMS-NBT]	0.2 to 2.0 mM procaine/GH3	O_2_^.–^**↓** procaine; **↓↓** GH3	Rusu and Lupeanu (1989) [22]
*In vivo* treatment	Rat tissue homogenates	20 mg procaine/kg body weight, 3 times/week × 9 weeks	O_2_^.–^**↓** lipid peroxidation **→** (liver, brain, kidney) **↓**SOD activity (brain) **↑**CAT activity (liver, kidney) Lipofuscin **↓** (brain, testicles, liver, heart)	Rusu et al. (1992) [23] Lupeanu (1999) [89] Radaceanu et al. (1991) [90]
	Rat tissue samples histopathological analysis			
*In vitro* sepsis	Neutrophils + LPS, triggered with fMLP	4.0 mM procaine	**↓** 50% LPS priming **↓** LPS-induced up-regulation, cytochrome b558	Jinnouchi et al. (2005) [64]
*In vitro* superoxide (O_2_^.–^) generation	Enzymatic system: [xanthine – XO – INT]	1.0 to 10.0 mM procaine/GH3	O_2_^.–^**↓** procaine; **↓↓** GH3	Gradinaru et al. (2009) [25]
*In vitro* total antioxidant capacity	ABTS cation + different local anesthetics	10 mM	Scavenging (%): tetracaine > procaine > lignocaine > benzocaine (99; 38; 21; 20%)	Librowski and Moniczewski (2010) [27]
*In vitro* ROS exposure	Isolated rabbit aortic rings	10^−5^M to 3 × 10^−3^ M procaine/lidocaine	Endothelium-dependent vasorelaxation **↑↓**	Lee et al. (2010) [26]
*In vitro* sepsis	Bovine aortic endothelial cells + IL-1*β*/LPS	10 mM procaine	**↓** NO production	Takaishi et al. (2013) [65]
*In vivo* and *in vitro* myocardial ischemia-reperfusion injury	Rat tissue samples	1,3,5-triazine-procaine derivatives	Cardioprotective **↓↓** ROS **↑** GSH, SOD, CAT, and GPx **↓** LOX-1 Antiapoptotic **↓** Bax; **↑** Bcl-2 Anti-inflammatory **↓**NF-*κ*B,	Qiang et al. (2019) [28]
	RAW264.7 macrophages transfected with NF − *κ*B + LPS	5 and 10 mg/mL, in K-H buffer solution x 45 min		
		100 mM		
*In vitro* ROS exposure	Jurkat cells + CuOOH	2.5 to 10 mM procaine/GH3	Membrane lipoperoxides **↓** procaine; **↓↓** GH3	Ungurianu et al. (2020) [29]
*In vitro* X-ray-DNA damage in human lymphocytes	PBMCs, young, and elderly subjects	0.25 to 10 mM procaine/GH3	Radioprotective (0.25 to 1 mM) GH3 **↓** DNA damage, young Procaine **↓** endogenous DNA strand breaks, aged Genotoxic (3 to 10 mM)	Ungurianu et al. (2020) [29]

ABTS: 2,2′-Azino-bis (3-ethylbenzothiazoline-6-sulfonic acid) diammonium salt; Bcl-2: B-cell lymphoma 2; Bax: Bcl-2-associated X protein; CAT: catalase; CuOOH: cumene hydroperoxide; fMLP: N-formyl-methionyl-leucyl phenylalanine chemotactic peptide; GH3: Gerovital H3; GPx: glutathione peroxidase; GSH: reduced glutathione; IL-1*β*: interleukin-1 beta; INT: 2-(4-iodophenyl)-3-(4-nitrophenol)-5-phenyltetrazolium chloride; LOX-1: lectin-like oxidized low-density lipoprotein receptor-1; LPS: lipopolysaccharide; NADH: reduced nicotinamide adenine dinucleotide; NBT: nitroblue tetrazolium; NF-*κ*B: nuclear factor kappa light chain enhancer of activated B cells; NO: nitric oxide; PBMCs: peripheral blood mononuclear cells; PMS: phenazine methosulfate; ROS: reactive oxygen species; SOD: Cu/Zn-superoxide dismutase; XO: xanthine oxidase.

**Table 2:** Molecular and cellular effects of procaine on mitochondria function, reported within *in vitro* studies.

Preclinic model	Target	Concentrations/doses	Relevant finding	Reference
*In vitro*	Rat liver mitochondria	0.5 to 10 mM procaine/GH3	Procaine (1 mM) **↑** basal state, respiration before ADP addition Procaine (>10 mM) **↓** 2,4-DNP, oxidative phosphorylation Lipid peroxidation **↓ ↓** procaine **↓** GH3	Tarba and Cracium, 1990 [33] Rusu (1990) [84] Borsa et al. (2002) [115] Ungurianu et al. (2020) [29]
*In vitro* cisplatin-induced nephrotoxicity	Rat renal cortical slices	2 mM procaine	**↓** mitochondrial injury **↓** cellular damage **↓** lipid peroxidation	Zhang and Lindup (1994) [86]
*In vitro* sepsis	Human vascular endothelial cells + LPS	0.01 to 1.0 mM procaine	**→** cell injury **↓** mitoKATP activation	de Klaver et al. (2006) [83]
*In vitro*	Rat dorsal root ganglion neurons	1, 5, and 10 mM procaine	**↑** depolarization of the mitochondrial membrane potential (*ΔΨ*m) **↑** [pH]m	Onizuka et al. (2010) [87]
*In vitro* neuroblastoma	Human cell line SH-SY5Y	12, 15, and 20 mM procaine	**↑** neurotoxicity, **↑** mitochondrial dysfunction, **↑** ROS **↑** lipid peroxidation **↑**DNA damage and apoptosis	Yu et al. (2017) [88]

2,4-DNP: 2,4-dinitrophenol; GH3: Gerovital H3; mitoKATP: mitochondrial ATP-sensitive potassium channel; ROS: reactive oxygen species.

**Table 3:** Molecular and cellular effects of procaine on lipoprotein oxidation and metabolism, reported within *in vitro* and *in vivo* studies, which support its antiatherogenic action.

Preclinic model	Target	Concentrations/doses	Relevant finding	Reference
*In vitro* and *in vivo* treatment	Human H295R adrenal cells, Hepa 1-6 mouse hepatoma cells	0.1, 1, 10, and 100 *μ*M procaine, for 48 h	**↓** steroidogenesis **↓** HMG-CoA reductase activity **↓** mRNA expression **↓** serum corticosterone	Xu et al. (2003) [35]
	Rats	25–100 mg procaine/kg body weight, 8 days		
*In vitro* treatment	Human, rat, dog plasma Rabbit aorta microsomes	1–5 mM procaine 0.25–0.50 mM procaine	**↓** plasma LCAT	Bell and Hubert (1980) [97] Bell (1981) [98]
			**↓** ACAT	
*In vitro* LDL oxidation	Human plasma LDL + Cu^2+^	0.1–1.0 mM procaine/GH3	Conjugated dienes **↓** procaine; **↓↓** GH3	Gradinaru et al. (2009) [25]
*In vitro* LDL oxidation	U937 macrophages + human plasma LDL + Cu^2+^	0.5–2.0 mM	TBARS **↓** procaine; **↓↓** GH3	Ungurianu et al. (2020) [29]
*In vitro* lipoprotein oxidation	Human serum lipoprotein concentrates	0.5 to 10 mM procaine/GH3	Lipid peroxidation: **↓** procaine; **↓↓** GH3	Ungurianu et al. (2020) [29]

ACAT: acyl-CoA cholesterol acyltransferase; GH3: Gerovital H3; HMG-CoA: 3-methylglutaryl-coenzyme A; LCAT: lecithin-cholesterol acyltransferase; LDL: low-density lipoproteins; TBARS: thiobarbituric acid reactive substances.

**Table 4:** Molecular and cellular effects of procaine reported within *in vitro* and *in vivo* studies, which support its neuroprotective actions.

Preclinic model	Target	Concentrations/doses	Relevant finding	Reference
*In vivo* treatment	Rat brain and liver mitochondria	60 mg procaine/kg body weight, 5 times/week × 4 weeks	MAO activity: **↓** procaine; **↓↓** GH3	Borsa et al. (2002) [115]
*In vitro β*-amyloid-induced neurotoxicity	Rat pheochromocytoma PC12 cells	1, 10, and 100 *μ*M procaine	**↓** amyloid peptide A*β*1-42 **↓** amyloid -induced ATP depletion **↓** glutamate neurotoxic effect	Lecanu et al. (2005) [116]
*In vivo* neuropathic pain	Rat tissue samples of L4-L6 spinal dorsal horn Behavior tests	2% procaine intrathecal injection in DMSO (10 *μ*L/kg)	**↓** JAK2 **↓**STAT3 (mRNA+protein) **↓** pain behavior	Li et al. (2016) [117]
*In vivo* MPTP-induced Parkinson's disease	Rat liver mitochondria Mouse brain homogenate Behavior tests	Procaine-imidazole derivatives 25, 50, and 100 mg/kg body weight, 3 days	**↓** MAO-B **↑** striatum antioxidant enzymes **↑** motor function	Wu et al. (2020) [37]

GH3: Gerovital H3; DMSO: dimethyl sulfoxide; JAK2: Janus kinase 2; MAO: monoamine oxidase; MPTP: 1-methyl-4-phenyl-1,2,3,6-tetrahydropyridine; STAT3: signal transducer and activator of transcription 3.

**Table 5:** Molecular and cellular effects of procaine as DNA demethylation and tumor-suppressive agent, reported within *in vitro* studies.

Preclinic model	Target	Concentrations/doses	Relevant finding	Reference
Breast cancer	Human MCF-7 cell line	0.005–0.5 mM procaine, 72 h	**↓** 40% DNA 5mC **↓***RARβ*2 gene CpG islands **↑** mitotic arrest	Villar-Garea et al. (2003) [38]
Lung and colon cancer	Human H460, A549, and HCT116 cells	2 mM procaine	**↑** silenced WIF-1	Gao et al. (2009) [130]
Hepatocellular carcinoma	Human hepatoma cells and nude mice bearing xenograft	1 mM procaine, 5 days	**↑** mitotic arrest **↓** CpG islands **↓** tumor volume	Tada et al. (2007) [131]
Leukemia	Human myeloid HL60 cells	Procaine analogues 0.5 mM, 72 h	**↓** DNMT1 **↓** CpG islands	Castellano et al. (2008) [132]
Bladder cancer	Human T24 and 5637 cells	5 – 10 *μ*M procaine	**↓** proliferation **↑** apoptosis **↔** APAF1 gene demethylation **↑** caspase-3/9; **↑** sFasL; **↓** MMP-9	Sun et al. (2012) [139]
Breast cancer	Human BT-20 (ER-negative) and MCF-7 (ER-positive) cell lines	Procaine and lidocaine, 0.01; 0.1, and 1 mM, 72 and 96 h	**↑** apoptosis **↓** DNA 5mC	Lirk et al. (2012) [144]
Lung cancer	Mouse lung cancer with A549 and NCI-H1975 xenograft	50 mg procaine/kg body weight × 3 weeks	**↓** tumor proliferation	Ma et al. (2016) [137]
	Human nonsmall cell lung cancer A549 and NCI-H1975 cell lines	100 nM procaine	**↓** cell proliferation **↓** EGFR mRNA	
Leukemia	Human NB4 cells	3–5 *μ*M procaine	**↑** CD11b, E-cadherin, G-CSF **↑** PPAR gamma **↔** chromatin remodeling—histone H3K4(Me)3 and H3K9Ac/S10P	Borutinskaite et al. (2016) [138]
Osteosarcoma	Human MG63 cells	2 *μ*M procaine	**↓** proliferation and migration **↑** apoptosis miR-133b upregulation AKT/ERK inactivation	Ying et al. 2017 [135]
Colon cancer	Human HCT11 cells	0.5, 1, 1.5, and 2 *μ*M procaine 3 *μ*M procaine + carboplatin 5 *μ*M procaine	**↓** proliferation and migration **↑** apoptosis **↑** RhoA expression **↑** DNA fragmentation **↓** DNA 5mC	Li et al. (2018) [136] Sabit et al. (2016) [145]
Mouse behavioral sensitization model	Peripheral blood cells	1–10 *μ*M procaine	**↓** DNMT3A mRNA	Anier et al. (2018) [39]
Gastric cancer	Human SGC-7901 and GES-1 cell lines	1–5 *μ*M procaine	**↓** DNMT1/DNMT3A activity **↓** proliferation **↑** apoptosis CDKN2A and RAR upregulation	Li et al. (2018) [136]
Molecular docking on B-DNA structures	Calf thymus	5–35 *μ*M procaine	**↔** binding to AT rich regions **→** electron donor to DNA bases	Ali et al. (2018) [133]
Breast cancer	Human MCF-7 cell line	5 *μ*M procaine	**→** anticancer activity	
Normal cells	Bovine skin fibroblasts	Procaine + SAH (1 mM)	**↓** DNMT1, DNMT3A, DNMT3B **↓** DNMT3B (procaine) **↑** TET3 dioxygenase	Schumann et al. (2020) [40]
Brain cancer	Human glioblastoma stem cells	5, 10, and 20 *μ*M procaine	**↓** survival and self-renewal **↓** ZDHHC15 transcripts **↓** GP130 palmitoylation **↓** activation of IL-6/STAT3	Fan et al. (2021) [41]

5mC: 5-methylcytosine; AKT: protein kinase B; APAF1: apoptotic peptidase activating factor 1; AT: adenine-thymine; CDKN2A: cyclin dependent kinase inhibitor 2A; CpG: 5′-cytosine-phosphate-guanine-3′; DNMT: DNA methyltransferase; EGFR: epidermal growth factor receptor; ERK: extracellular signal-regulated kinase; ER: oestrogen receptor; G-CSF: granulocyte colony-stimulating factor; GP130: glycoprotein 130; H3K4 (Me)3: tri-methylation at the 4th lysine residue of the histone H3 protein; H3K9Ac/S10P: phospho-acetylated histone H3; miR: microRNA; MMP-9: matrix metallopeptidase 9; PPAR: peroxisome proliferator-activated receptor; RAR: retinoic acid receptor; RhoA: Ras homolog family member A; SAH: S-adenosyl-L-homocysteine; sFasL: serum soluble fas ligand; STAT3: signal transducer and activator of transcription 3; TET3: translocation enzyme-3; WIF-1: Writ inhibitory factor-1; ZDHHC15: zinc finger Asp-His-His-Cys-type palmitoyltransferase 15.

## Data Availability

Review article based on PubMed database.
